# Four reflections on the new global mental health priorities

**DOI:** 10.1017/S2045796019000726

**Published:** 2019-11-19

**Authors:** José Miguel Caldas-de-Almeida

**Affiliations:** Lisbon Institute of Global Mental Health, Comprehensive Health Research Centre, Nova Medical School, Nova University of Lisbon, Rua do Instituto Bacteriológico, no 5, 1150-190 Lisboa, Portugal

**Keywords:** Community mental health, mental health, psychiatric services, rights of persons with disabilities

## Abstract

The discussion of the achievements and limitations of the strategies prioritised in global mental health that has taken place in recent years contributed to a unified vision for action that addresses the gaps still existing on prevention, treatment, quality of care and human rights protection. This editorial presents four reflections on the impact of this vision on the definition of future priorities, particularly in the areas of policy implementation, services reconfiguration and organisation, human rights and research. It concludes that further debate is needed to redefine the balance between priorities and strategies that can better promote an effective response to the needs of low and middle income countries, and to ensure an efficient coordination of efforts in the future.

## Introduction

Defining priorities is one of the most important challenges in global mental health. Given the magnitude of mental health problems and the gaps that still exist in this area, it is of utmost importance to concentrate all efforts and resources on the objectives and strategies that may more effectively contribute to attaining the main goals of global mental health.

This new field, particularly in the last 20 years, registered an enormous growth and had significant achievements in the inclusion of mental health in the global health agenda, the development of innovative knowledge and practices, the training of leaders and professionals and the support to policy development. This growth led to the consolidation of a global mental health movement that is now highly visible worldwide, but, at the same time, it also brought to light a growing diversity of perspectives on what should constitute the core of global mental health and how its various strategies should be prioritised.

In the last few years, a number of important contributions to this debate have been made by several authors, who raised questions such as the definition and content of global mental health, the risks of an excessive influence of the western biomedical approach, the balance between the focus on common mental disorders and the focus on severe mental illnesses, the balance between prevention, treatment and care, the real impact of global mental health initiatives on the improvement of mental health in low resource countries, future priorities in research and the level of attention dedicated to the reform of mental institutions (Summerfield, 2008; Bracken *et al*., 2016; Freeman, 2016; Patel, 2016; Saraceno and Barbui, 2016; Saxena, 2016; Chatterjee, 2017; Cohen and Minas, 2017, among others).

Several institutional initiatives have also directly or indirectly contributed to this discussion: among others, the Grand Challenges in Global Mental Health (Collins *et al*., 2011), the WHO Global Mental Health Plan (World Health Organisation, 2013), the Gulbenkian Global Mental Health Platform (World Health Organization and Calouste Gulbenkian Foundation, 2017) and the Lancet Commission on Global Mental Health and Sustainable Development (Patel *et al*., 2018).

The Lancet Commission, while highlighting the importance of the achievements of global mental health, recognised the need for a discussion on the limitations of its principles and strategies, and proposed a reformulation and expansion of the global mental health agenda.

The proposed agenda resulted in great part from the recognition that, to reduce the global burden of mental disorders, it is necessary to go beyond reducing the treatment gap, to also address the gaps on prevention, quality of care and human rights.

It also confirmed the importance of life course and human rights approaches. Additionally, it included among the key priorities the provision of adequate community-based care to people with long-term mental disorders, and highlighted the importance of ‘the reconfiguration of care away from hospitals and into community settings’, explicitly stating that the closing of psychiatric hospitals should be started in low income countries, consolidated in middle income countries and completed in high income countries.

The fact that this new agenda includes all these components in a unified vision for action represents a significant reinforcement of global mental health. This is amplified by the fact that this new vision results from the convergence of advances made in the various scientific disciplines relevant to mental health, and from the international consensus reached on the key importance of the values focused on the respect for human rights, the recovery approach and the participation of people with mental disorders in mental health policy and delivery.

In fact, as shown by the Lancet Commission, it was the convergence of knowledge from epidemiology, social sciences, neurosciences and clinical and services research that made it possible: (1) to understand the importance of investing in prevention and of prioritising interventions during childhood and adolescence; (2) to recognise the limitations of the categorical approach in diagnosis and the need to complement it with a dimensional approach and (3) to value the importance of structuring mental health systems in a way that enhances the opportunities for intervention at all stages of the evolution of mental health problems, from well-being to different stages of the disease (Patel *et al*., 2018).

On the other hand, it was, in great part, the consensus reached on the key values of mental health care that gave new prominence to the fact that, although psychiatric institutions are widely acknowledged to be among the main sites of human rights abuses, the truth is that issues of reform of mental institutions have not received the attention they deserve from global mental initiatives (Chatterjee, 2017; Cohen and Minas, 2017).

This new framework for action will certainly have important consequences on the definition of priorities. In this editorial I will present four reflections on these consequences.

## First reflection

In the last decade, significant progress was made on the elaboration/updating of national mental health policies. However, neither in low nor high resource countries, has this been translated into significant implementation. As pointed out by the Lancet Commission, when it comes to mental health all countries are developing countries (Patel *et al*., 2018).

Thus, to help countries making the mental health system reforms that are indispensable to significantly improve the mental health of their populations, global mental health should place at the head of its priorities the development of countries’ policy implementation capacities.

The barriers to implementation are known: lack of political commitment, insufficient financial resources, resistance to change, challenges to implementation of mental health care in primary-care settings, insufficient number of workers who are trained and supervised in mental health care and frequent scarcity of public-health perspectives in mental health leadership (Saraceno *et al*., 2007).

Therefore, at the global level, it will be necessary to continue the efforts made to include mental health among the public health agenda: increasing coordination with other global movements (sustainable development, chronic diseases, human rights, wellbeing and climate change); strengthening alliances involving different stakeholders (people with experience of mental disorders, families, professionals, academia, NGOs and international organisations) and ensuring an effective coordination of efforts, benefiting from the unique contribution the World Health Organisation can give in this domain.

It will be also necessary to increase the commitment of donors: most low income countries allocate a very limited proportion of their already limited health budgets to mental health, and are significantly dependent on external support to initiate a mental health reform process and to fund the costs of the transition phase.

At the national level, generating and strengthening political commitment deserves much more attention than it has received in the past. Although it is largely recognised that it's one of the major obstacles to the improvement of mental health all over the world, the truth is that there is no clear guidance on how to generate and strengthen political commitment to mental health development, and this problem has received surprisingly little research attention so far (Caldas de Almeida and Minas, 2014).

Much more attention will also have to be paid to the improvement of leadership and governance in countries. In the last decade, a significant number of international courses have been created for leaders and people conducting innovative programmes in mental health policy and services, which have provided important contributions in this area. Yet, many more training activities should be organised to respond to all the needs, and incentives should be created to enhance the involvement in teaching activities of people from countries that have been particularly successful in the implementation of mental health policies (Lund *et al*., 2014).

Other priorities include initiatives that may develop countries’ technical capacities in: (a) the implementation, monitoring and evaluation of mental health plans; (b) improvement of information systems and (c) development of new financing models facilitating the reallocation of resources and the creation of incentives aligned with the strategic changes that are required.

International support is also needed to strengthen users’ associations and to empower them as partners in all steps of mental health policy and its implementation.

## Second reflection

Given the specific characteristics of mental health care, scaling up of services is not enough to ensure the advances that are needed; a reconfiguration of the mental health system and a reorganisation of services are always indispensable.

In the last decades, we have significantly increased our knowledge on how to prevent mental disorders, integrate mental health care in primary care, provide effective community based mental health care and inpatient treatment in general hospitals and ensure the deinstitutionalisation and social inclusion of people with long-term mental disorders (World Health Organization and the Global Mental Health Platform, 2014).

Examples of reforms that made possible a reorganisation of services, based on these principles, exist not only in countries with a high level of resources but also in low resource countries (Caldas de Almeida and Horvitz-Lennon, 2010; dos Santos *et al*., 2016). However, despite these advances, most countries continue to face serious deficiencies in the prevention of mental disorders and in the delivery of mental health care.

The incorporation in the routine practice of the innovative concepts and models of task shifting, task sharing, collaborative care and integration of mental health in chronic diseases care still is very limited in most countries.

Progress in shifting away from care based on large psychiatric institutions to community-based services has been very uneven across countries, and in many places the number of mental hospitals remains very high and still consumes much of the resources allocated to mental health. Most importantly, in most countries there is still much to be done to ensure the provision of a person-centred, recovery-focused mental health service, with the capacity to offer people with severe mental disorders good quality clinical and social care, as well as helping them to have meaningful activities and to sustain or create strong social networks in their communities.

Finally, the predominant approach in the development of mental health care worldwide continues to ignore the evidence that most mental disorders represent variable clusters of trans-syndromal symptom dimensions that cannot be captured by a simple categorical diagnosis (Van Os *et al*., 2019); and the evidence that a significant part of the effects of treatment interventions depends not on the technical aspects of the interventions, but on the setting and characteristics of the service and on relational aspects of treatment, is similarly ignored (Bracken *et al*., 2016; Van Os *et al*., 2019).

How can global mental health address these problems in a more effective way?

The establishment of schemes of technical assistance to countries interested in implementing mental health service reforms should be a key priority. As proved in several global mental health initiatives (Caldas de Almeida and Cohen, 2008; Chatterjee, 2017), the use of international consultants to work with the authorities and local services, providing technical support and conducting training activities, proved to be a very effective strategy, especially if articulated with international mechanisms of financial support. Initiatives contributing to promote the dissemination of good practices and diminish the resistances to change that still exist among professionals, policy makers and the public should also be promoted. In the research area, studies that may increase our knowledge on the most effective strategies to improve the restructuring of mental health systems and the reorganisation of high quality services in countries of low, medium and high levels of resources are particularly needed.

## Third reflection

Human rights have been one of the major components of global mental health since the beginning, and were responsible for a significant number of the most important advances registered in mental health in the last decades. Much more can be expected from the human rights approach in the future, but to fully benefit from this potential, it will be necessary to address several important issues such as: (a) clarification of the ambiguities that persist on the definition of and relations between the concepts of mental disorders, mental disabilities and psychosocial disabilities, which represent a serious obstacle to the incorporation of the Convention on the Rights of Persons with Disabilities principles into the mental health law of many countries (Caldas de Almeida, 2019); (b) evaluation of the real impact of violations of the human rights of persons with mental disorders; (c) development and incorporation in services of strategies to reduce the use of coercion and compulsory treatment and (d) development of concerted actions aiming at completing the replacement of psychiatric institutions and ensuring the right to live independently in the community.

## Fourth reflection

Research has made many valuable contributions to the progress of the global mental field. After the overview of the research priorities by the Grand Challenges Initiative (Collins *et al*., 2011) and the recent contribution of Patel (2016), in which he describes the potential contribution of this field to discovery science, there is not much to add on this issue.

For this reason, I will just leave two brief notes. The first one to highlight the special importance of investing in studies contributing to: (a) understanding the effects of differences in the organisation and delivery of national mental health-care systems on mental health of people with mental disorders; (b) assessing the cost-effectiveness of different models of financing, organising and providing mental health care and (c) increasing our knowledge on the effects of the service-related factors and the relational aspects of interventions on outcomes, and on how to incorporate the new knowledge in the routine practice. The second one to stress the importance of actions that could strengthen research centres in low resource countries and provide practical support to researchers from these countries in access to grants and mentorship, and in publishing papers.

## Conclusions

The debate carried out in the last few years on the goals and strategies of global mental health gave important contributions to the redefinition of priorities in this field, grounded on the advances made at the scientific level and on the international agreement on the importance of the values associated with the human rights and recovery approaches. Further debate is now needed to redefine the balance between the different priorities and strategies that can give a better response to the needs of low and middle income countries and to ensure an efficient coordination of efforts in the future, taking advantage of the unique contribution the World Health Organisation can provide in this domain.
